# Effect of Supplementation with Black Chokeberry (*Aronia melanocarpa*) Extract on Inflammatory Status and Selected Markers of Iron Metabolism in Young Football Players: A Randomized Double-Blind Trial

**DOI:** 10.3390/nu15040975

**Published:** 2023-02-15

**Authors:** Błażej Stankiewicz, Mirosława Cieślicka, Jan Mieszkowski, Andrzej Kochanowicz, Bartłomiej Niespodziński, Andrzej Szwarc, Tomasz Waldziński, Joanna Reczkowicz, Elżbieta Piskorska, Miroslav Petr, Anna Skarpańska-Stejnborn, Jędrzej Antosiewicz

**Affiliations:** 1 Institute of Physical Education, Kazimierz Wielki University, Jana Karola Chodkiewicza 30, 85-064 Bydgoszcz, Poland; 2 Department of Human Physiology, Nicolaus Copernicus University, Karłowicza 24, 85-092 Bydgoszcz, Poland; 3 Department of Gymnastics and Dance, Gdansk University of Physical Education and Sport, Kazimierza Gorskiego 1, 80-336 Gdansk, Poland; 4 Faculty of Physical Education and Sport, Charles University, 162 52 Prague, Czech Republic; 5 Department of Team Sports, Gdansk University of Physical Education and Sport, Kazimierza Gorskiego 1, 80-336 Gdansk, Poland; 6 Faculty of Health Sciences, Łomża State University of Applied Science, Akademicka 14, 18-400 Lomza, Poland; 7 Medical Department of Bioenergetics and Physiology of Exercise, Faculty of Health Sciences, Medical University of Gdansk, Dębinki 1, 80-211 Gdansk, Poland; 8 Department of Pathobiochemistry and Clinical Chemistry, Nicolaus Copernicus University Collegium Medicum, M. Curie Skłodowskiej 9, 85-094 Bydgoszcz, Poland; 9 Department of Morphological and Health Sciences, Faculty of Physical Culture in Gorzow Wielkopolski, Poznan University of Physical Education, Orląt Lwowskich 4–6, 66-400 Gorzów Wielkopolski, Poland

**Keywords:** inflammation, reactive oxygen species, supplementation, black chokeberry extract, iron

## Abstract

The use of herbal medicinal products and supplements in amateur and professional sports has increased in the last decades. This is because most of these products and supplements contain bioactive compounds with a variety of biological properties that exert a physiological effect on the human body. The aim of this study was to analyze the effect of dietary supplementation with lyophilized black chokeberry extract on the levels of pro-inflammatory cytokines, hepcidin, and selected markers of iron metabolism in a group of young football players. This double-blind study included 22 male football players (mean = 19.96 ± 0.56), divided into two groups: supplemented and placebo. Before and after a 90-day period of training combined with supplementation (6 g of lyophilized black chokeberry extract), participants performed maximal multistage 20-m shuttle run tests at the beginning and at the end of the supplementation period, with blood sampled for analysis at different times before and after exercise. The levels of IL-6, IL-10, ferritin, myoglobin, hepcidin, 8-OHdG, albumin, and TAC were analyzed. The analysis of variance revealed a significant effect of 90-day supplementation with the lyophilized extract on changes in the IL-6 and IL-10 levels, and TAC induced by maximal aerobic effort. In conclusion, supplementation with lyophilized black chokeberry extract improves the performance and antioxidant status of serum in humans and induces protective changes in inflammatory markers.

## 1. Introduction

In recent years, the use of dietary interventions and herbal supplements by athletes has tremendously increased. That is because such interventions and supplements are increasingly available and, while they improve the physical performance of athletes, they are advertised as “substances not prohibited in sport”. Further, supplements from herbs or plants may enhance muscle growth and fat burning, or possess anti-inflammatory, antiviral, and antibacterial properties [1,2,3]. On the other hand, the consumption of herbal supplements may contribute to a decrease in physiological disorders that occur as a result of physical activity [3].

Regular intensive physical activity is accompanied by many physiological changes, such as dysregulation of the pro-oxidant–antioxidant balance, hyperthermia, hypoglycemia, hemolysis, and increased hepcidin expression [3,4,5,6,7]. Exercise-induced stress can increase free radical formation, which can be determined by body iron stores. For example, regular training reduced ferritin levels (body iron stores), which was associated with lower oxidative stress [8]. The sources of oxidant production during exercise are not well established. However, there is increased data indicating that there is an interrelationship between oxidative stress, iron, inflammation, and skeletal muscle weakness and fatigue [4,9].

Oxidative stress is related to increased inflammation, which is strictly associated with iron metabolism [10]. Exercise-induced increased levels of pro-inflammatory cytokines, such as IL-6, stimulate hepcidin biosynthesis, which blocks duodenal iron absorption and its liberation from hepatocytes and macrophages into the bloodstream [11]. The ensuing limitation of the labile iron pool reduces oxidative stress and inflammation caused by physical activity [4]. 

Most herbal products that are defined as “dietary supplements” contain concentrated juice; lyophilized or water/alcohol extracts of seeds, gums, roots, leaves, bark, berries, or flowers; and a number of phytochemicals, such as carotenoids and polyphenols, including phenolic acids, alkaloids, flavonoids, glycosides, saponins, and others [12,13]. However, there are no specified thresholds for the qualitative and quantitative composition of such products. Irrespective of the marketing of processed natural herbal supplements that apparently improve health and physical performance, some plants used for supplement production may contain doping substances, and some products based on herbal extracts may be contaminated or adulterated by agents prohibited in sports. Consequently, unprocessed herbal products (such as juice, concentrate, and lyophilizate) obtained from well-known plants with a proven therapeutic effect and prepared in a way that does not involve long-term processing are of particular interest to athletes. 

Chokeberry (*Aronia melanocarpa*) contains more phenolic compounds than most other blackberries, is beneficial to health, and has well-documented antioxidative properties [12,13,14]. Hence, the use of chokeberry supplements in sports could be beneficial, in that they could affect not only the antioxidant state but also reduce post-exercise inflammation [12,13]. Nonetheless, there is a dearth of studies on the anti-inflammatory effect of black chokeberry products, and their effect on hepcidin levels and selected markers of iron metabolism, especially in the context of sports performance. Therefore, there is a need to verify these effects, and to establish the extent to which dietary supplementation with polyphenol-rich lyophilized black chokeberry extract may alter the inflammatory indices of elite athletes and influence the changes in iron metabolism elicited by extensive and intense exercise loads.

We hypothesized that an adequate intake of a lyophilized extract of black chokeberry may affect post-exercise plasma levels of IL-6 and IL-10, and changes in iron metabolism parameters, and could be used to maintain the optimal antioxidant status in exercising well-trained individuals.

## 2. Materials and Methods

### 2.1. Ethics

This study was conducted following the approval of the Collegium Modicum Bydgoszcz Bioethics Commission (consent No. KB 382/2017]). All researchers involved in the study have respected the principles of the Helsinki Declaration. Prior to the study, the participants gave written informed consent to participate in the study, with the option to withdraw at any time for any reason.

### 2.2. Study Design

The study was designed as a double-blind randomized controlled trial with parallel groups. The supplementation protocol involved a 90-day (7 times per week) supplementation program with 6 g of lyophilized black chokeberry extract in semi-professional football players of the MUKS Zawisza Bydgoszcz Soccer Club (Bydgoszcz, Poland). During the initial study visit, the subjects’ ages, body compositions, and heights were recorded. All participants were examined by a professional physician and were healthy. Before and after 90 days of supplementation, the participants completed the maximal multistage 20-m shuttle run test. Further, before and after the supplementation period, venous blood samples were taken four times for serum analysis. Before the study, the lyophilized black chokeberry extract used during the supplementation was analyzed by antioxidant testing (Section 2.3).

### 2.3. Antioxidant Testing of Black Chokeberry Extract

The lyophilized black chokeberry extract was analyzed by antioxidant tests at the Lubuskie Center of Agrotechnical Innovation and Implementation of the University of Zielona Góra (Zielona Góra, Poland). For the tests, 2,2′-azino-bis(3-ethylbenzthiazoline -6-sulfonic acid) (ABTS), 2,2-di-phenyl-1-picrylhydrazyl radical (DPPH), and other reagents were purchased from Sigma Aldrich (St. Louis, MO, USA). Ultrapure water (Milli-Q) was used in all analyses (Millipore, Bedford, MA, USA).

#### 2.3.1. DPPH Antioxidant Test

DPPH antioxidant testing was performed as described by Pownall*,* et al. [15]. Briefly, 500 μL of 100 μM DPPH solution in methanol was mixed with 100 μL of sample dilutions and 400 μL of 50 mM phosphate buffer (pH 7.0). The blank consisted of 400 μL of the buffer, 100 μL of sample dilution, and 500 μL of methanol. The control sample was prepared by mixing 500 μL of each of the phosphate buffer and DPPH solution. All samples were incubated for 30 min at room temperature. Sample absorbance was then measured (λ = 515 nm) using a Shimadzu UV-1603 spectrophotometer (Tokyo, Japan). DPPH radical scavenging activity (%) was calculated as [(A517cA517s)/A517c]100, where “c” and “s” represent the absorbance of the control and sample, respectively. The results are expressed as IC50 values, and refer to the concentration required to scavenge 50% of DPPH free radicals. Each sample was analyzed three times. The coefficient of variation was always less than 5%. 

#### 2.3.2. ABTS Antioxidant Test

Antioxidant activity was determined by a colourimetric assay using the cation radical of ABTS, as described by Re*,* et al. [16]. The ABTS cation radical was generated in K2O8S2 solution. Phosphate buffer (PBS, pH = 7.0) was used for sample dilution. Sample absorbance was measured at 734 nm. The results are expressed in equivalents of μmol Trolox/cm^3^. The coefficient of variation was always less than 5%.

The results of DPPH and ABTS analyses of the lyophilized black chokeberry extract used in the current study are presented in Table 1. 

### 2.4. Participants

Participants (*n* = 22) were semi-professional football players and members of the MUKS Zawisza Bydgoszcz Soccer Club (Bydgoszcz, Poland) playing in the Junior Central League. Before study enrollment, they were screened by laboratory assistants with respect to the following exclusion criteria: positive history of morphological or cardiac problems, significant orthopedic problems, or intake of ergogenic nutritional supplements (e.g., creatine, β-hydroxy-β-methylbutyrate, etc.) or anabolic/catabolic hormones (e.g., androstenedione, dehydroepiandrosterone, etc.) within 6 months prior to the study. The subjects were randomly assigned to the supplemented (*n* = 10) or control groups (*n* = 12) using an online randomization software (http://www.graphpad.com/quickcalcs/index.cfm (accessed on 8 February 2023) [17]. They were asked to adopt a similar eating pattern during the entire study, based on a diet individually specified for their age group and the intensity of physical activity. The characteristics of each group are provided in Table 2.

### 2.5. Supplementation

Both groups completed a 90-day (7 times per week) supplementation program. The experimental population was taking 6 g of lyophilized black chokeberry extract (a dose within a margin of dietary safety). 

The control group received the same number of identical-looking rice-flavored gelatin capsules as the supplemented group. The lyophilized black chokeberry extract and placebo supplements were both produced by MLB (Biotrade, Poznan, Poland), and placed in identical plastic boxes marked in code indicating the type of preparation and recommended dose.

The margin of dietary safety was determined in pilot studies involving 45 young physically active men (mean age = 19.96 ± 0.56 years), performed before the start of the current study. In the pilots, no gastrointestinal disturbances associated with the dose used herein were observed. 

### 2.6. Exercise Protocol

Study subjects performed maximal aerobic effort (MAE) by a maximal multistage 20 m shuttle run test [17]. During testing, all participants ran back and forth on a 20 m course and touched the 20 m line. Throughout the test, a sound signal was emitted from a prerecorded tape. The frequency of the sound signals increased by 0.5 km·h^−1^ each minute from a starting speed of 8.5 km h^−1^. When the subject could no longer follow the pace, the last stage number was recorded and used to predict the maximal oxygen uptake (VO_2max_). On the testing days (before and after the supplementation period), the air temperature was 18.3 °C and 18.5 °C, respectively, with 57% and 59% humidity, respectively. All participants were wearing indoor sports footwear and tracksuits. The test–retest reliability coefficient for testing is approximately 0.95 for adults. 

### 2.7. Blood Collection and Analysis

Blood samples were taken for analysis at four points in time: immediately before and after MAE, and 6 and 24 h after MAE. The blood was collected into 5 mL tubes (Sarstedt) that contained an anticoagulant. The serum was separated by centrifugation and aliquoted into 500 μL portions. Then, the samples were frozen in liquid nitrogen and stored at −80 °C until further analysis. 

To assess the baseline and altered iron (FE) levels, plasma was collected into tubes with lithium heparin at two points in time: immediately before and after MAE. It was then analyzed using an in vitro IRON 2 (Roche/Hitachi Cobas c.) system and a Cobas C analyzer 501 (Hitachi, Tokyo, Japan).

Biochemical analyses of serum ferritin, myoglobin, hepcidin, IL-6, and IL-10 levels were performed using commercially available highly sensitive enzyme-linked immunosorbent assay kits from DRG International Inc. (841 Mountain Ave, Springfield, NJ 07081, USA) and a Thermo Fisher Scientific ELISA Analyzer (Thermo Fisher Scientific, Waltham, MA, USA). Total antioxidative capacity (TAC) was measured using TAC Fast Track ELISA kit no. LDN-DM P-4100 (Omnignostica Forschungs GmbH, Austria) and 8-OHdG levels were measured using the Human 8-OHdG ELISA kit (Shanghai SunRed Biological Technology Co. Ltd., No. 6497, Shanghai, China). 

Lactate concentration was measured in capillary blood collected from the earlobe before and immediately after MAE, using a Dr. Lange Plus LP20 biochemical analyzer.

### 2.8. Statistical Analysis

Data were analyzed using a set of two two-factor mixed-design analyses of variance (ANOVA). In the first analysis, the within-subject factor (repeated measures) represented the supplementation effect (before and after) on the performance variables during MAE and biochemical parameters at rest, while the between-subject factor represented the group effect (supplemented or control). In the second analysis, the within-subject factor (repeated measures) represented the effort effect of MAE (baseline, immediately post MAE, and 6 h and 24 h post MAE) and the between-subject factor represented the group effect (supplemented or control). Significant interaction between factors was subsequently analyzed using Tukey’s post-hoc test. The reliability of assumptions of this statistical test was checked using the Shapiro–Wilk test for normality, and Levene’s test for the homogeneity of variance. Further, the effect size was estimated by eta-squared statistics (ƞ^2^). Values equal to or greater than 0.01, 0.06, and 0.14 indicated a small, moderate, and large effect, respectively. The significance threshold was set at *p* < 0.05. The required total sample size of 12 (ANOVA: 2 × 4) and 16 participants (ANOVA: 2 × 2) was estimated with G*Power software ver. 3.1.9.4. (Franz Faul et al., Universität Kiel, Kiel, Germany) for a large effect size, and a power of 0.80. Due to the fact that the same training lasted for over the 90 days in both supplemented and control groups representing the same sport club, the number of available players was limited; thus, only the large effects could be detected. Statistical analysis was performed using Statistica 13 software (TIBCO Software Inc., Palo Alto, CA, USA).

Statistical analysis was performed using Statistica 13 software (TIBCO Software Inc., Palo Alto, CA, USA).

All participants recruited for the study completed the study with no adverse events reported. The results of MAE before and after supplementation with the lyophilized black chokeberry extract are presented in Table 3. Statistical analysis (ANOVA) revealed a significant increase in the length of the distance run and the delta change of lactate levels in the supplemented group. No significant changes in the MAE performance variables before and after supplementation in the control group (*p* > 0.05) were apparent.

The results of the analysis of serum levels of inflammatory markers at rest before and after the supplementation are presented in Table 4. 

**Figure 1:**
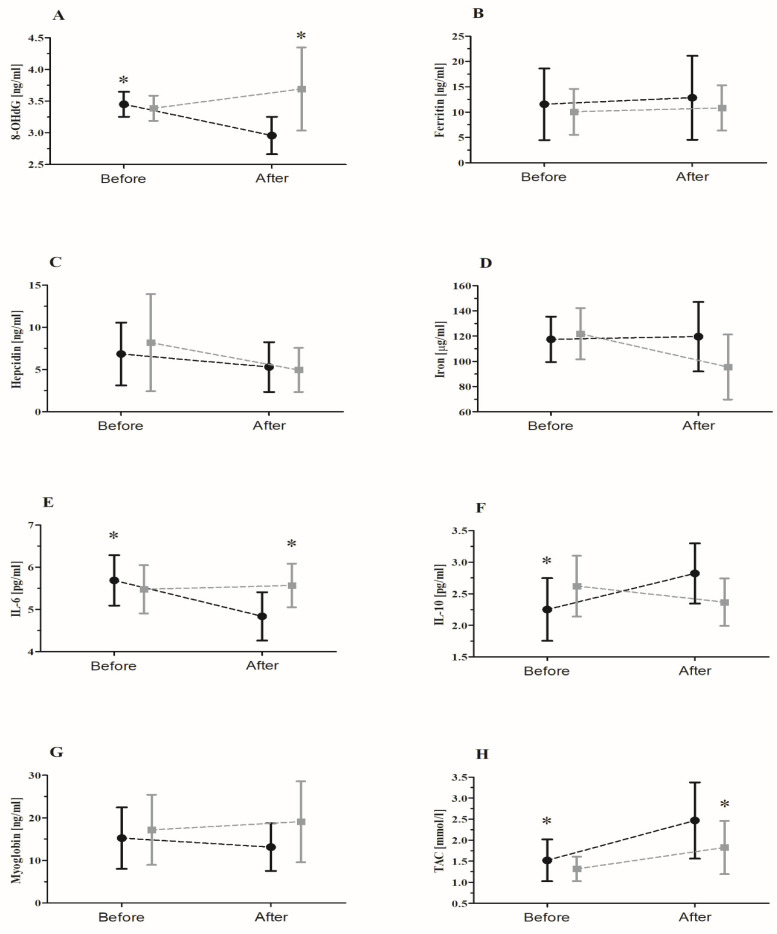
The mean ± SD resting levels of specific inflammatory markers before and after 90 days of supplementation with a lyophilizate of black chokeberry extract: (**A**) 8-OHdG, (**B**) ferritin, (**C**) hepcidin, (**D**) iron, (**E**) IL-6, (**F**) IL-10, (**G**) myoglobin, and (**H**) TAC. Black circles, supplemented group (*n* = 12); gray squares, placebo group (*n* = 10). Abbreviations: 8-OHdG, 8-hydroxydeoxyguanosine; IL-6, interleukin 6; IL-10, interleukin 10; TAC, total antioxidant capacity; * significant difference vs. supplemented group after 90 days of supplementation with a lyophilizate of black chokeberry extract.

Statistical analysis (ANOVA) revealed a significant effect of interaction (supplementation × group) on the resting values of 8-OHdG, IL-6, IL-10, and TAC (Figure 1). There was a 14.5% decrease in 8-OHdG levels (*p* < 0.05) and a 15.0% decrease in IL-6 levels (*p* < 0.01) in the supplemented group. By contrast, the resting levels of IL-10 (20.0%, *p* < 0.01) and TAC (85.2%, *p* < 0.01) significantly increased after supplementation with the lyophilized black chokeberry extract.

Before the supplementation, statistical analysis (ANOVA) of inflammatory marker levels revealed a significant effect of MAE on 8-OHdG (*p* < 0.01), IL-6 (*p* < 0.01), IL-10 (*p* < 0.01), myoglobin (*p* < 0.01), and TAC (*p* < 0.01). The greatest increase from baseline was noted for the levels of 8-OHdG immediately after exercise (38.3%, *p* < 0.01); IL-6 (83.9%, *p* < 0.01) and IL-10 (121.5%, *p* < 0.01) 6 h after exercise; and myoglobin 24 h after exercise (50.9%, *p* < 0.01). By contrast, TAC decreased immediately after exercise (37.0%, *p* < 0.01). No effect of any of the analyzed factors on ferritin and hepcidin levels was noted.

After the supplementation, statistical analysis (ANOVA) revealed a significant effect of interaction (effort × group) on changes in the IL-6 and IL-10 levels, and TAC induced by MAE (Table 5). 

**Figure 2:**
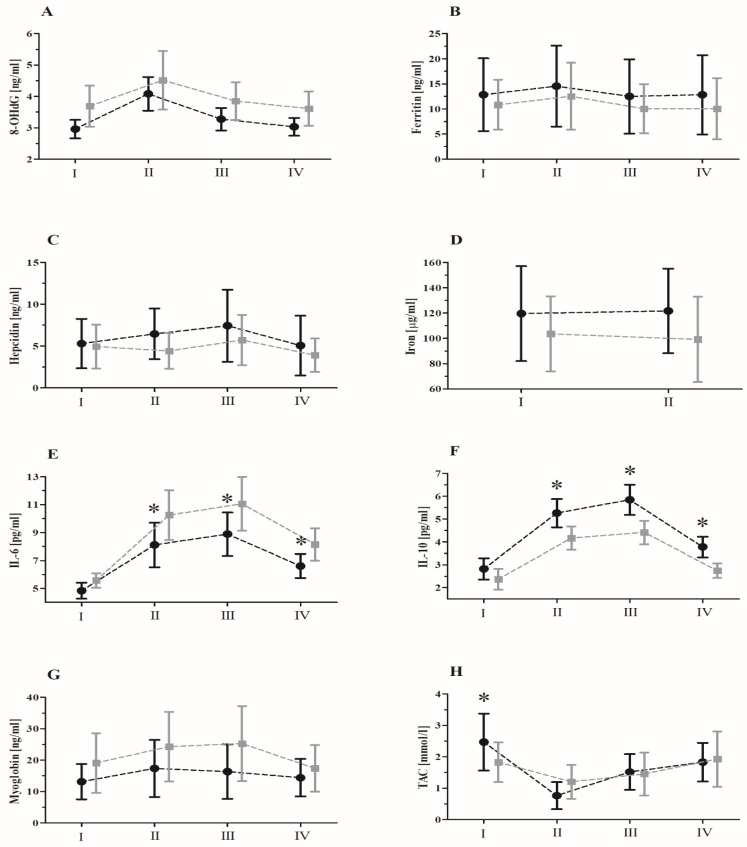
The mean ± SD values of serum levels of specific inflammatory markers induced by maximal aerobic effort (MAE) after 90 days of supplementation with a lyophilizate of black chokeberry extract: (**A**) 8-OHdG, (**B**) ferritin, (**C**) hepcidin, (**D**) iron, (**E**) IL-6, (**F**) IL-10, (**G**) myoglobin, and (**H**) TAC. Black circles, supplemented group (*n* = 12); gray squares, placebo group (*n* = 10). Abbreviations: I, baseline; II, immediately after MAE; III, 6 h post MAE; IV, 24 h post MAE; 8-OHdG, 8-hydroxydeoxyguanosine; IL-6, interleukin 6; IL-10, interleukin 10; TAC, total antioxidant capacity; * significant difference vs. placebo at the particular time point.

Post hoc analysis revealed that IL-6 levels immediately after (31.3%, *p* < 0.01) and 6 h after exercise (35.9%, *p* < 0.01) were significantly lower in the supplemented group than in the control group. Conversely, serum IL-10 levels immediately after exercise (20.0%, *p* < 0.01) and 6 h after exercise (24.5%, *p* < 0.01) in the supplemented group were significantly higher than those in the control group (Figure 2).

Further, as before the supplementation, a significant effort effect of MAE on 8-OHdG and myoglobin levels was noted, regardless of the group. Finally, TAC significantly decreased immediately after MAE, regardless of the group effect, and the levels before exercise were higher in the supplemented group than those in the control group, as was also noted for the values at rest after the supplementation period.

## 3. Discussion

In the current study, we tested the hypothesis that a 90-day (7 times per week) supplementation program with 6 g of lyophilized black chokeberry extract affects the adaptation process and exercise-induced inflammation and exerts beneficial effects on the selected parameters of iron metabolism. We focused mainly on the interaction between the supplementation and changes in the serum levels of LA, and selected markers of inflammation (IL-6 and IL-10), oxidative stress (8-OHdG), antioxidant potential (TAC), iron metabolism (iron, ferritin, hepcidin), and skeletal muscle damage (myoglobin). We show that administration of a lyophilized black chokeberry extract leads to an increase in the serum TAC and IL-10 levels, and a decrease in IL-6 levels during the recovery period after maximal aerobic testing

The effectiveness of the tested supplementation program was reflected by a significant post-supplementation increase in the resting serum TAC and a decrease in 8-OHdG levels (Figure 1). 

In the supplemented group, lactate levels after MAE were significantly higher than those in the placebo group, which may suggest an improvement of anaerobic metabolism as a result of training and supplementation. Higher post-exercise lactate concentrations after the supplementation period indicate that athletes’ exercise tolerance increased. Similar results were found by Pilaczynska-Szczesniak*,* et al. [18]. 

In the current study, the effect of MAE on young football players was reflected by a post-exercise decrease in the serum TAC, which was observed both prior to and after the supplementation (Figure 2). While the observed changes did not reach statistical significance in either case (supplementation or control), the trends are similar to those reported by Groussard, et al. [19] and Skarpańska-Stejnborn, Basta, Sadowska, and Pilaczyńska-Szcześniak [6]. A decrease of TAC perturbs free radical neutralization, which could increase lipid peroxidation, biological membrane dysfunction, and intercellular oxidation [20]. It may also lead to changes in the redox status of iron and the activation of many other inflammatory pathways, resulting in increased oxidative stress and inflammation [21]. 

While TAC increased after the supplementation, we also observed a decrease in resting 8-OHdG levels, which remained low after MAE in the supplemented group compared to placebo. 8-OHdG is one of the predominant forms of ROS-induced DNA lesions and, thus, is a commonly used biomarker of oxidative stress [22]. In a previous study, Stankiewicz*,* et al. [23] found that 7 weeks of supplementation with black chokeberry juice did not alter the 8-OHdG levels and TAC before and after similar maximal exercise (a 20 m shuttle run test). The difference in outcomes could be related to the length of the supplementation period (90 days vs. 49 days, current vs. prior study, respectively), the form of the supplement (6 g of lyophilized extract vs. 200 mL of juice, current vs. prior study, respectively), and group size (*n* = 22 vs. *n* = 20, current vs. prior study, respectively).

It is notable that flavonoid extracts, such as black chokeberry lyophilizate, are rich in flavonoids, α-tocopherol, and ascorbic acid. These substances protect against increased oxidative stress and suppress free radical generation. Furthermore, they interact with each other. On the one hand, high flavonoid concentrations have a protective effect on oxidative changes facilitated by ascorbic acid. On the other hand, high concentrations of ascorbic acid positively interact with flavonoid activity [24]. Hence, the high levels of antioxidants that the study group was supplemented with protected the participants against damage from reactive oxygen radicals and affected extracellular inflammation. 

In the current study, we also showed that the favorable changes observed during the supplementation period are associated not only with the changes in TAC but also with changes in many inflammatory markers, such as IL-6 and IL-10. IL-6 plays an important role in the regulation of inflammation. Typically, physical exercise is accompanied by an increase in IL-6 levels. We observed this effect in the current study in both the supplemented and control groups. Importantly, supplementation with the lyophilized black chokeberry extract reduced the increase in IL-6 levels for up to 24 h after exercise. To date, this effect has been reported only rarely [6]. According to one in vitro study, black chokeberry extract suppresses the STAT3/IRF1 pathway by inhibiting the phosphorylation of STAT3, and thus decreases the expression of IL-6 [25]. This, in turn, stimulates fatty acid mobilization and regulates the inflammatory process [6]. 

In the current study, supplementation with the lyophilized black chokeberry extract affected the resting levels of IL-6. Unlike Skarpańska–Stejnborn et al., who reported that there is no effect of proposed supplementation on IL-6 [6], we observed a significant effect of the Aronia lyophilisate on IL-6 levels (Figure 1). However, the changes were not significantly correlated with hepcidin levels, both prior to and after the supplementation. The observed trends may indicate an association between those proteins as, typically, IL-6 induces hepcidin synthesis in hepatocytes [26].

Flavonoids maintain the balance between oxidized, reduced, and radical forms of antioxidants, which is an important element of protection against increased ROS levels. In the current study, in addition to affecting IL-6 levels, the tested supplementation protocol rich in flavonoids affected serum levels of IL-10, another inflammatory marker. IL-10 is an anti-inflammatory cytokine whose blood levels increase after exercise. Accordingly, we here observed a significant increase in IL-10 levels after MAE. The effect was more pronounced in football players in the supplemented group than in those in the control group. Notably, in the supplemented group, the IL-6 level increase was smaller than that in the control population. IL-6 release from the exercising muscle is accompanied by the appearance of IL-10 and other anti-inflammatory cytokines in the circulation. The presented observations indicate that the lyophilized chokeberry extract induces an anti-inflammatory response independent from IL-6 [27].

According to Aaseth and Stoa Birketvedt [28], prolonged aerobic physical activity may lead to a post-exercise increase in plasma iron levels. The main underlying mechanism is hemolysis and rhabdomyolysis, associated with increased inflammation. Accordingly, chelation of free iron as a control mechanism of iron metabolism after intensive exercise is important, considering the pro-inflammatory potential of iron [29]. Flavonoids, the major biological compounds of black chokeberry lyophilizate, harbor a hydroxyl group on the C-ring and hence may chelate free iron to reduce its concentration [30]. In the current study, we did not observe any changes in the markers of iron metabolism at rest and after MAE. That may be because the employed aerobic testing method, despite its specificity, did not lead to hemolysis and rhabdomyolysis. Similar observations were made by Antosiewicz, Kaczor, Kasprowicz, Laskowski, Kujach, Luszczyk, Radziminski, and Ziemann [29] and Skarpańska-Stejnborn, Basta, Sadowska, and Pilaczyńska-Szcześniak [6]. The above findings may indicate that the specific aerobic testing intensity of exercise in well-adapted populations should be adjusted toward increased overloading. On the other hand, the findings could also indicate high physical preparedness for exercise of the studied group of football players.

The current study has some potential limitations. The first limitation is the relatively small sample size. Further studies should be performed, with an increased sample size and/or implementing a crossover study design. Second, the football players’ field position or volume of competition play (starter vs. non-starter) was not considered during randomization. Third, the study did not include a control of the dietary regime, and potential changes in the amount of consumed fruits and/or vegetables rich in flavonoids might have interfered with the applied intervention. In addition, we used an indirect VO_2max_ evaluation method. Nonetheless, that procedure was only used to cause cellular response and observe post-exercise inflammation. Finally, most of the presented results were obtained using ELISA. This is a well-known and widely used method for the analysis of numerous compounds; however, measurement errors are possible. Hence, highly sensitive and specific LC–MS/MS methodology should be used in future studies. 

## 4. Conclusions

In this study, we showed that the administration of a lyophilized black chokeberry extract, which possesses strong antioxidant activity, leads to an increase in the serum TAC and IL-10 levels and a decrease in IL-6 levels during the recovery period after maximal aerobic testing. Hence, the consumption of a lyophilizate of natural black chokeberry extract may result in beneficial effects, reducing the consequences of an intensive training load. The presented findings justify the use of lyophilized black chokeberry extract as a dietary supplement for athletes undergoing MAE, and can be a starting point for considering its use to enrich the dietary patterns of populations other than athletes.

## Figures and Tables

**Table 1:** The antioxidant potential of lyophilized chokeberry extract used in the current study.

Method	Group Division
	Antioxidant Potential [μmol Trolox/cm^3^]
DPPH	172.4 ± 0.7
ABTS	211.1 ± 0.61

Abbreviations: ABTS, 2,2′-azino-bis (3-ethylbenzthiazoline-6-sulphonic acid); DPPH, 2,2-diphenyl-1-picrylhydrazyl.

**Table 2:** The characteristics of participants before and after supplementation with a lyophilizate of black chokeberry extract (mean ± SD).

Variable	Supplemented (*n* = 10)			Control (Placebo) (*n* = 12)		
	Before	After	*p*	Before	After	*p*
Age (years)	19.86 ± 0.61	–	–	20.05 ± 0.52	–	–
Body height (cm)	180.00 ± 4.65 *	–	–	176.12 ± 4.21	–	–
Body mass (kg)	68.86 ± 6.49	67.54 ± 6.17	0.63	64.86 ± 5.46	65.12 ± 6.19	0.91
Body mass index	21.27 ± 1.75	21.04 ± 1.66	0.75	20.63 ± 1.38	20.85 ± 1.44	0.71
Fat mass (%)	10.80 ± 1.39	10.52 ± 1.34	0.64	9.55 ± 2.43	9.67 ± 2.53	0.90

* Significant difference vs. control group at *p* < 0.05 before supplementation with a lyophilizate of black chokeberry extract.

**Table 3:** Participant performance in maximal multistage 20 m shuttle run test before and after supplementation with a lyophilizate of black chokeberry extract (mean ± SD).

Variable	Supplemented (*n* = 12)		Control (Placebo) (*n* = 10)		Effect	*p*-Value
	Before	After	Before	After		
Distance (km)	2.50 ± 0.20	2.82 ± 0.18 ^#^	2.47 ± 0.32	2.59 ± 0.19	GR RM GR × RM	0.21 0.01 ** 0.01 *
Lactate (mM) delta change	8.24 ± 1.71	9.47 ± 1.85 ^#^	9.00 ± 1.72	7.79 ± 1.53	GR RM GR × RM	0.49 0.98 0.01 **

Abbreviations: GR, group; RM, repeated measure. ^#^ Significant difference before and after training at *p* < 0.05, significant difference at * *p* < 0.05 and ** *p* < 0.01.

**Table 4:** Two-factor ANOVA with repeated measures (2 groups × 2 time points) of the resting serum levels of inflammatory markers before and after 90 days of supplementation with a lyophilizate of black chokeberry extract.

Variable	Effect	F	df	*p*-Value	Effect Size (η^2^)	Post-hoc Outcome
8-OHdG	GR RM GR × RM	11.80 0.55 1.73	1, 20 1, 20 1, 20	<0.01 ** 0.46 <0.01 **	0.37 0.02 0.32	S < P S_before_ > S_after_ S_after_ < P_after_
Ferritin	GR RM GR × RM	0.47 0.97 0.06	1, 20 1, 20 1, 20	0.50 0.33 0.80	0.02 0.04 < 0.01	
Hepcidin	GR RM GR × RM	0.02 0.01 2.92	1, 20 1, 20 1, 20	0.86 0.90 0.10	<0.01 <0.01 0.12	
Iron	GR RM GR × RM	0.37 <0.01 0.94	1, 20 1, 20 1, 20	0.55 0.99 0.34	0.02 <0.01 0.06	
IL-6	GR RM GR × RM	1.36 15.08 23.06	1, 20 1, 20 1, 20	0.25 <0.01 ** <0.01 **	0.06 0.43 0.53	Before > After S_before_ > S_after_ S_after_ < P_after_
IL-10	GR RM GR × RM	0.21 1.89 21.41	1, 20 1, 20 1, 20	0.64 0.18 <0.01 **	0.01 0.08 0.51	S_before_ < S_after_
Myoglobin	GR RM GR × RM	1.84 0.01 1.77	1, 20 1, 20 1, 20	0.18 0.94 0.19	0.08 <0.01 0.08	
TAC	GR RM GR × RM	6.88 28.87 7.24	1, 20 1, 20 1, 20	0.02 * <0.01 0.01 *	0.25 0.59 0.26	S > P Before > After S_before_ < S_after_ S_after_ > P_after_

Abbreviations: 8-OHdG, 8-hydroxydeoxyguanosine; IL-6, interleukin 6; IL-10, interleukin 10; TAC, total antioxidant capacity; GR, group; RM, repeat measure; S, supplemented group; P, placebo group; significant difference at * *p* < 0.05 and ** *p* < 0.01.

**Table 5:** Two-factor ANOVA with repeated measures (2 groups × 4 time points) † of the serum levels of inflammatory markers induced by maximal aerobic effort (MAE) after 90 days of supplementation with a lyophilizate of black chokeberry extract.

Variable	Effect	F	df	*p*-Value	Effect Size (η^2^)	Post-Hoc Outcome
8-OHdG	GR RM GR × RM	7.25 72.54 1.26	1, 20 3, 60 3, 60	0.01 <0.01 * 0.29	0.26 0.78 0.05	S < P II, III > I, IV; II > III
Ferritin	GR RM GR × RM	0.55 3.32 0.26	1, 20 3, 60 3, 60	0.46 0.06 0.85	0.02 0.11 0.01	
Hepcidin	GR RM GR × RM	2.74 0.38 0.55	1, 20 3, 60 3, 60	0.11 0.76 0.64	0.12 0.02 0.02	
Iron	GR RM GR × RM	1.84 0.10 0.52	1, 20 3, 60 3, 60	0.19 0.75 0.47	0.09 0.01 0.02	
IL-6	GR RM GR × RM	24.27 190.65 10.32	1, 20 3, 60 3, 60	<0.01 * <0.01 * <0.01 *	0.54 0.90 0.34	S < P I < II, III, IV; II < III; III > IV S-II < P-II; S-III < P-III;
IL-10	GR RM GR × RM	57.57 572.43 16.43	1, 20 3, 60 3, 60	<0.01 * <0.01 * <0.01 *	0.74 0.96 0.45	S > P I < II, III, IV; II < III; III > IV S-II > P-II; S-III > P-III; S-IV > P-IV
Myoglobin	GR RM GR × RM	3.92 8.56 1.71	1, 20 3, 60 3, 60	0.06 <0.01 * 0.17	0.16 0.29 0.07	II, III > I, IV
TAC	GR RM GR × RM	6.42 26.09 4.39	1, 20 3, 60 3, 60	0.02 <0.01 * <0.01 *	0.24 0.56 0.18	S > P I > III, IV > II, S-I > P-I

Abbreviations: 8-OHdG, 8-hydroxydeoxyguanosine; IL-6, interleukin 6; IL-10, interleukin 10; TAC, total antioxidant capacity; GR, group; RM, repeat measure; S, supplemented group (*n* = 12); P, placebo group (*n* = 10); I, baseline; II, immediately after MAE; III, 6 h post MAE; IV, 24 h post MAE; * significant difference at *p* < 0.01. † This does not apply to iron. In the case of iron, ANOVA with repeated measures involved 2 groups and 2 time points (baseline and immediately post MAE).

## Data Availability

Datasets analyzed during the current study will be available at the end of the project of which they are part (Grant from the National Science Centre, Poland—number 2020/37/B/NZ7/01794).
